# Categorization of the Histopathological Diagnosis of Breast Core Needle Biopsies and the Correlation of the Risk of Malignancy With Diagnostic Accuracy

**DOI:** 10.7759/cureus.103866

**Published:** 2026-02-18

**Authors:** Poorni B Thiruvarasu, Sneha Kulkarni

**Affiliations:** 1 Histopathology, Sri Devaraj Urs Medical College, Kolar, IND

**Keywords:** atypical ductal hyperplasia, breast, breast carcinoma in situ, breast neoplasms, carcinoma, diseases category, ductal

## Abstract

Introduction: Core needle biopsy (CNB) is used to detect carcinoma of the breast, but it does not always provide a definitive diagnosis. Cases can be categorized into diagnostic categories to facilitate further management decisions. The United Kingdom’s National Health Service Breast Screening Program uses five “B categories” for reporting CNBs of the breast. These categories can help bring uniformity to the reporting of biopsies.

Materials and methods: Biopsy cores were assigned to one of the five B categories (B1-B5). Biopsy diagnoses were then correlated with the diagnoses from the resection specimens. Sensitivity (SN), specificity (SP), positive predictive value (PPV), negative predictive value (NPV), and diagnostic accuracy (DA) were calculated.

Results: CNBs from 66 cases were collected and categorized as either B1, B2, B3, B4, or B5. Three of the cases (4%) were categorized as B1, eight cases (12%) were categorized as B2, four cases (6%) were categorized as B3, a further four cases (6%) were categorized as B4, and one case (1%) was categorized as B5 carcinoma in situ, while 46 cases (65%) were categorized as invasive. All four of the B3 lesions involved atypical intraductal epithelial proliferations. The SN, SP, PPV, NPV, and DA for the cases categorized as malignant B4 and B5 were 88.8, 90.9, 94.1, 83.3, and 89.6, respectively.

Conclusion: More so than reports that are merely descriptive, reports that include B categorization help convey and bring uniformity to pathologists’ perspectives, especially in uncertain cases. Categorization thus facilitates communication between pathologists and clinicians and, therefore, the guidance of patient management.

## Introduction

Breast carcinoma is the most common malignancy affecting females, accounting for 25.1% of all cancers, and is the second most common cause of cancer deaths after lung cancer [1]. Higher rates of mortality are reported in females in developing nations than in developed nations [1]. Core needle biopsy (CNB), in particular, Tru-Cut biopsy, is one of the definitive investigations in the diagnosis of carcinoma of the breast and is considered superior to fine needle aspiration cytology because it can be used for histological grading and hormonal studies and, thus, assists in determining the appropriate treatment for a patient [2]. However, in some cases, the findings may be uncertain for a particular diagnosis. Such cases can be assigned to diagnostic categories to facilitate further management decisions.

The United Kingdom’s National Health Service Breast Screening Program (NHSBSP) suggests five B categories for the reporting of Tru-Cut breast biopsies [3], based on which categories of breast lesions can be properly identified. The NHSBSP guidelines have been incorporated into the guidelines for nonoperative diagnostic procedures and reporting in breast cancer screening provided by the Royal College of Pathologists [4]. This categorization reduces errors resulting from under- or over-diagnosis of lesions and allows pathologists to express uncertainty in definitive diagnoses. The categories thus contribute to uniformity in the reporting of biopsies and the assessment of the risk of malignancy (ROM) and diagnostic accuracy (DA) for various categories. Similar categorizations are widely accepted and used to report fine needle aspiration for breast cytology.

At present, no definitive categorization is used in the reporting of breast CNBs at our institution. Thus, in this study, these biopsies were categorized according to the B categories proposed by the NHSBSP, and the ROM and DA for each category were determined.

## Materials and methods

We conducted a retrospective observational study in the pathology department from January 2022 to December 2024. The study was approved by the institutional ethical committee prior to its commencement (Approval Number: SDUAHER/KLR/R&D/CEC/F/NF/66/2024-25). The study included all patients who underwent breast CNB. Thus, regarding the study population, all females were included who underwent incisional biopsies of the breast with or without subsequent lumpectomy or mastectomy. Any patient who underwent prior chemotherapy or hormone therapy was excluded from the study. Regarding sample size, for a specificity of 54.8% or higher with a precision of 10 and a confidence interval of 95%, 60 participants were required, with a 10% sampling error [2].

Hematoxylin and eosin slides of the biopsies were assessed by two pathologists. The biopsy cores were assigned to one of the NHSBSP’s five B categories (B1-B5) [3]. The B1 category includes lesions assessed as unsatisfactory/normal tissue only. The B2 category includes lesions that are benign. The B3 category includes lesions with or without epithelial atypia for which the malignancy potential is uncertain. Lesions suspicious for malignancy were included in the B4 category. The B5 category includes three subcategories of malignancy: type a (in situ); type b (invasive); and type c (not assessable). The diagnoses based on the biopsies were then correlated with the diagnoses based on the resection specimens. The histological diagnosis of the subsequent lumpectomy or mastectomy, when available, was assessed and correlated.

In the statistical analysis, data were entered into a Microsoft Excel data sheet (Microsoft Corporation, Redmond, WA) and were analyzed using IBM SPSS Statistics version 22 (IBM Corp., Armonk, NY). We calculated the sensitivity (SN), specificity (SP), positive predictive value (PPV), negative predictive value (NPV), and diagnostic accuracy (DA) while maintaining the corresponding lumpectomy/mastectomy report as the gold standard. The number of malignant cases confirmed by lumpectomy or mastectomy divided by the total number of cases in the diagnostic category served to calculate the ROM.

## Results

In total, the study included 66 cases. The patients were between 22 and 69 years of age, with most in their fifth decade of life. The left breast was more commonly involved than the right breast, and most of the cases occurred in the left upper outer quadrant of the breast. The patients commonly presented with a lump in the breast. A few also presented with pain, while some were asymptomatic. The demographic details are summarized in Table 1.

**Table 1:** Demographic data of the study population. UI, upper inner quadrant; UO, upper outer quadrant; LO, lower outer quadrant; LI, lower inner quadrant; NAC, nipple areolar complex.

Demographic data	Groups	Number of cases
Age (years)	<25	4
	25-40	12
	40-60	28
	>60	22
Laterality	Right	25
	Left	41
Site	UI	8
	UO	23
	LO	19
	LI	15
	NAC	1
Presenting symptoms	Asymptomatic	16
	Lump	35
	Pain	15
	Nipple discharge	1

**Figure 1:**
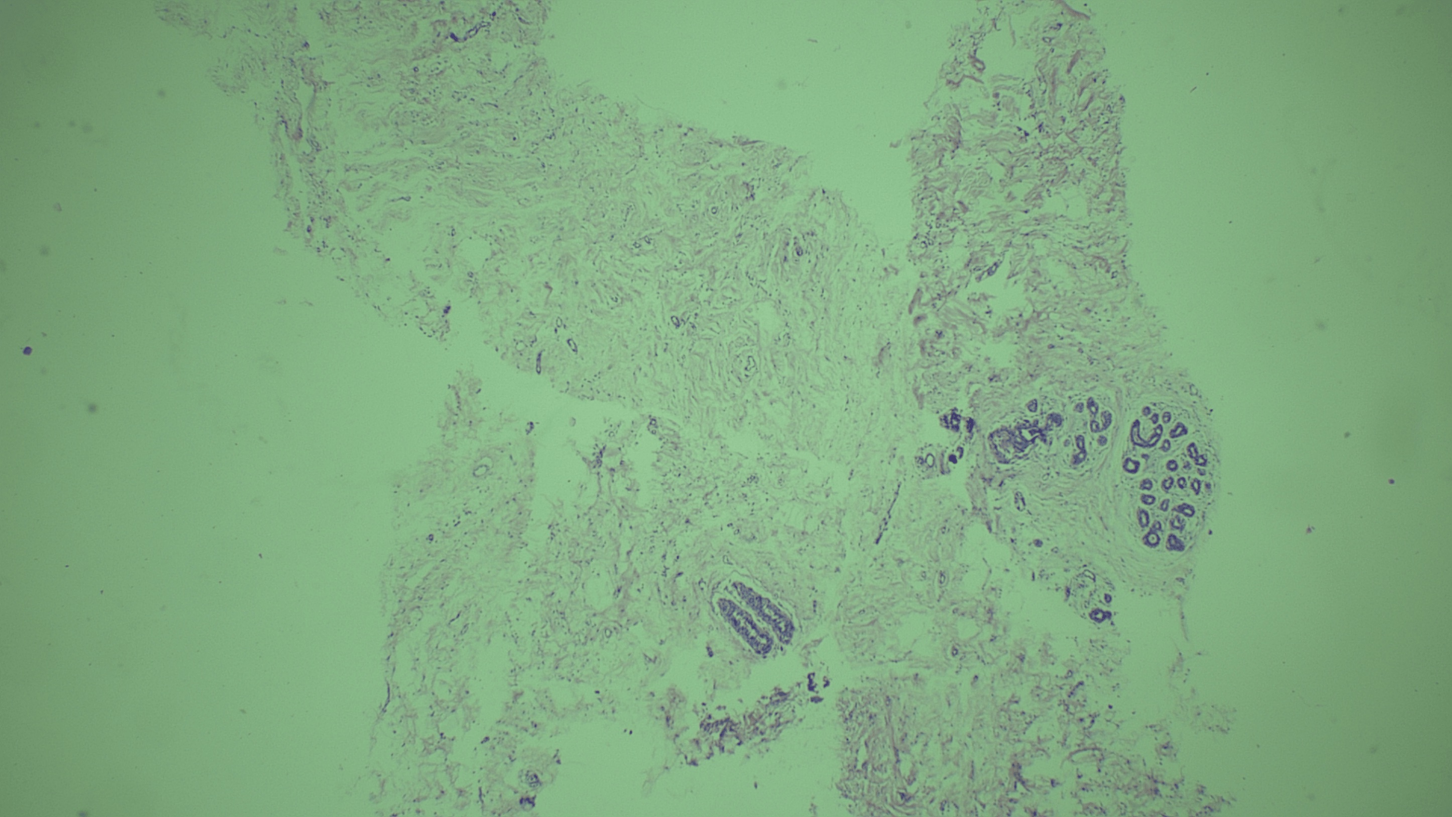
Only fibrocollagenous stroma with few ducts - B1 (hematoxylin and eosin stain, 40x magnification).

**Figure 2:**
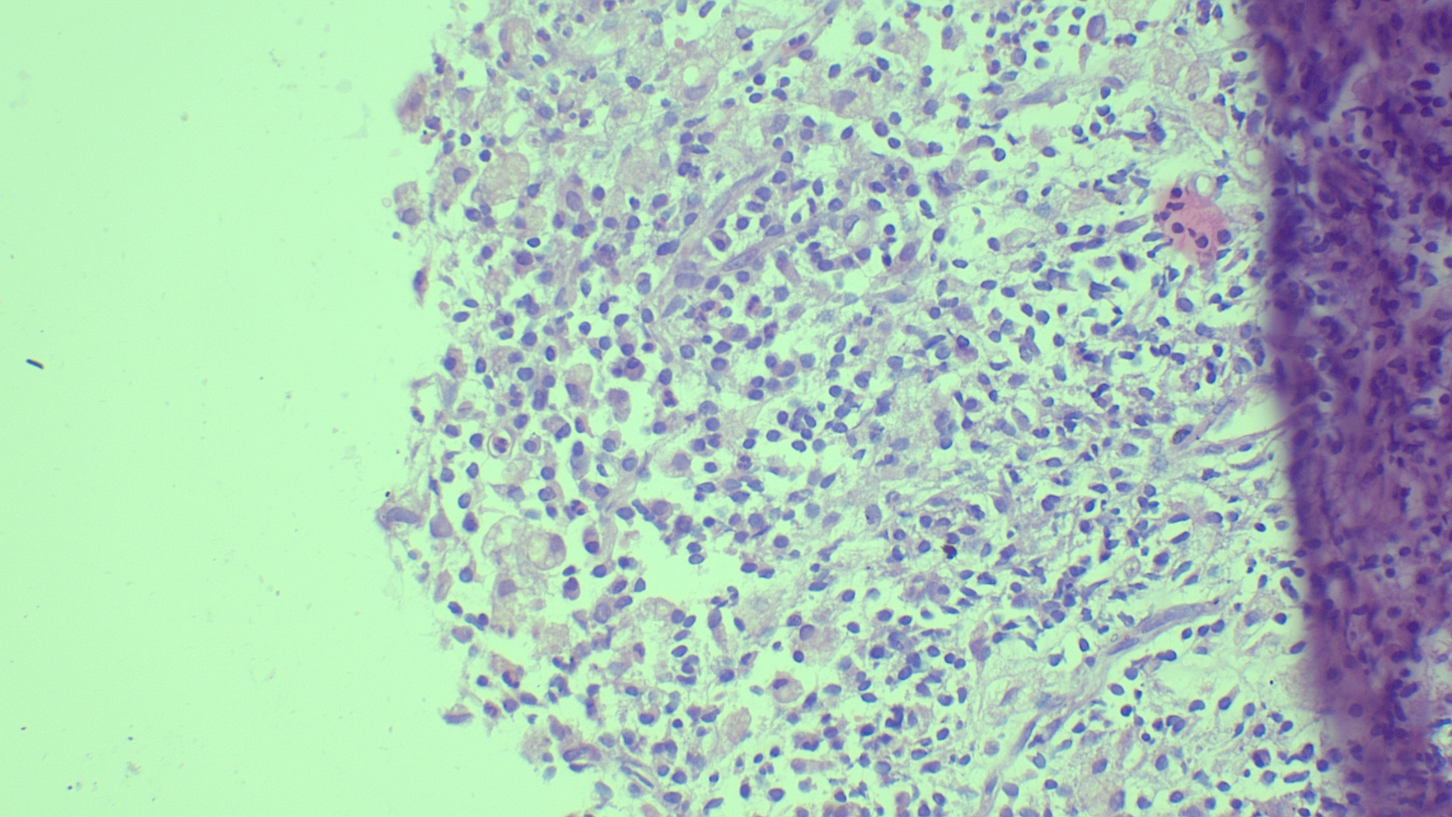
Stroma with inflammatory infiltrate including lymphocytes and plasma cells - B2 (hematoxylin and eosin stain, 100x magnification).

**Figure 3:**
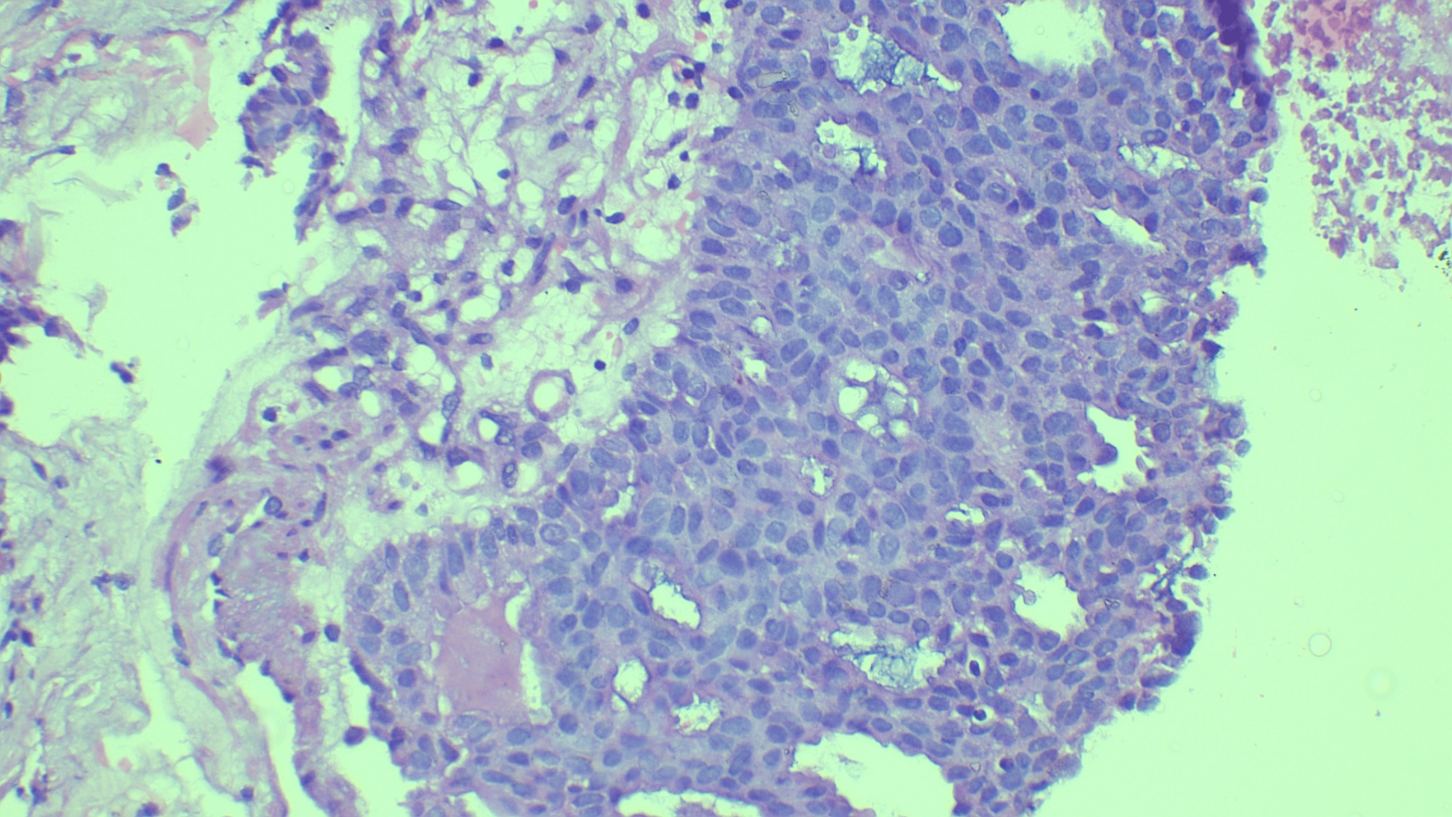
Intraductal proliferations forming cribriform and solid areas of more than 2 mm in area - B3 (hematoxylin and eosin stain, 100x magnification).

**Figure 4:**
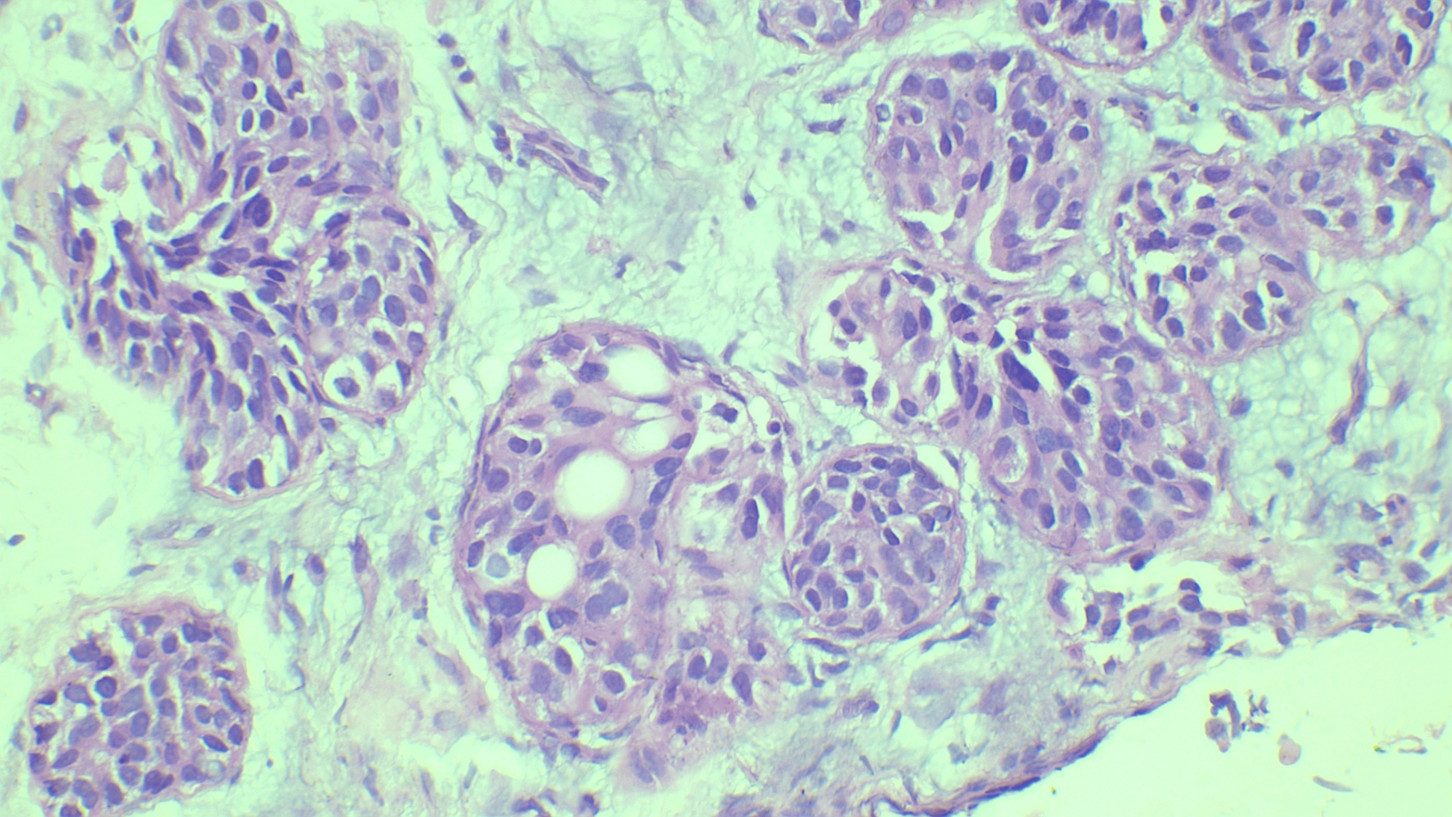
Intraductal proliferations forming complete solid areas given as ductal carcinoma in situ - B5a (hematoxylin and eosin stain, 400x magnification).

**Figure 5:**
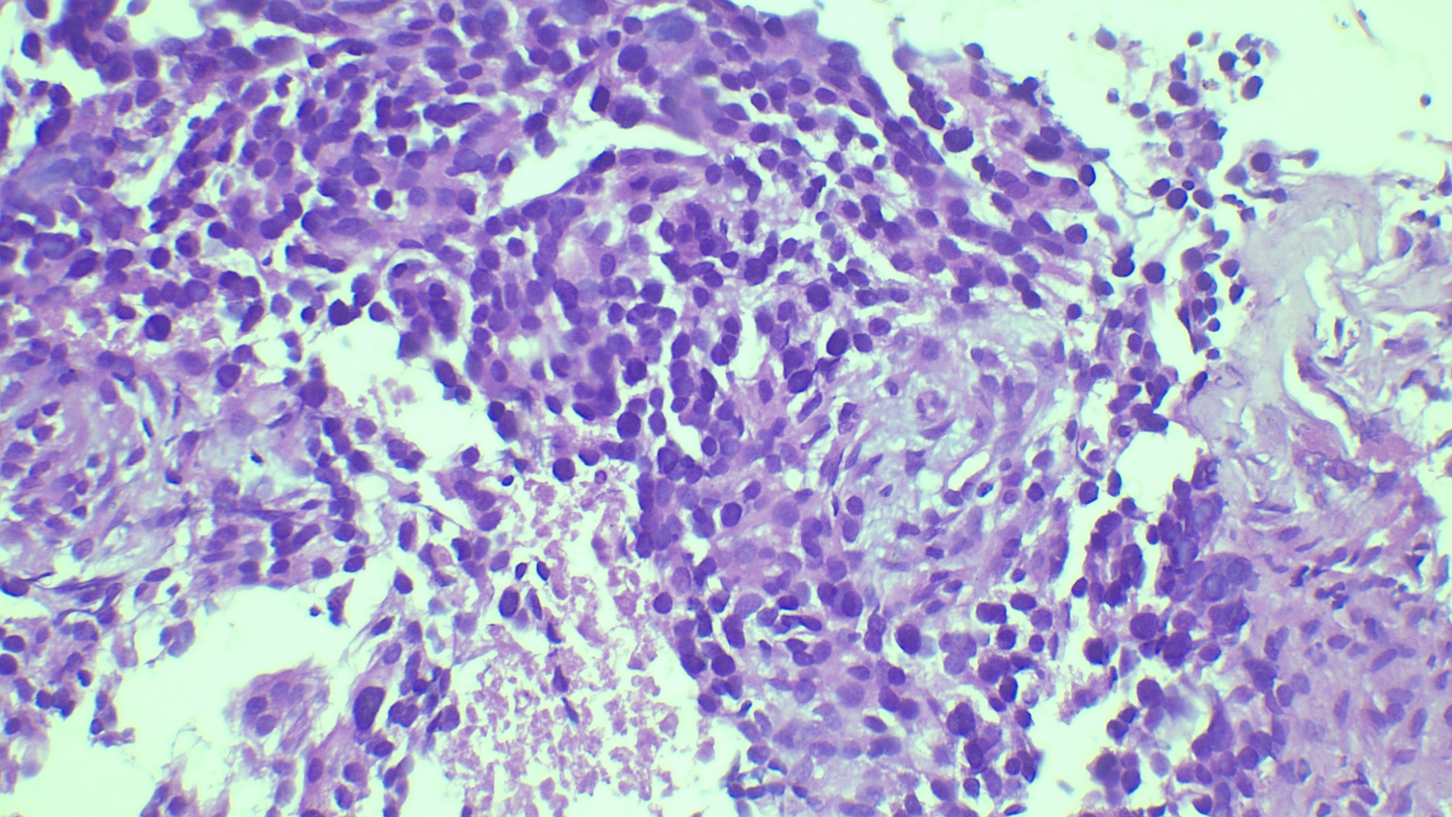
Highly pleomorphic tumor cells seen invading the stroma, given as invasive ductal carcinoma - B5b (hematoxylin and eosin stain, 400x magnification).

We assigned the 66 cases to the B categories as follows. Three cases (4%) were categorized as B1, two of which showed only fibrocollagenous stroma, while the third showed only fibrotic tissue. Figure 1 shows a case of fibrosis categorized as B1. Eight cases (12%) were categorized as B2, of which three were fibroadenomas, a further three were benign phyllodes, one was diagnosed as a chronic granulomatous lesion, and one involved an inflammatory lesion. Figure 2 shows a case of inflammation categorized as B2. Four cases (6%) were categorized as B3, all of which showed atypical intraductal epithelial proliferations (AIDEPs). Figure 3 shows a case of AIDEP. Forty-seven cases (65%) were categorized as B5 malignant, of which one (1%) was in ductal carcinoma in situ (DCIS). Figure 4 shows the case of DCIS. Forty-five cases (65%) were infiltrating ductal carcinomas (IDCs), and one was malignant phyllodes. Figure 5 shows an IDC case.

The distribution of the cases into the various categories is summarized in Table 2.

**Table 2:** Distribution of the cases into the various categories with details of the individual cases. CNB, core needle biopsy; AIDEP, atypical intraductal epithelial proliferations; IDC, infiltrating ductal carcinoma; DCIS, ductal carcinoma in situ.

Category	Number of cases	Diagnosis on CNB
B1	2	Fibrocollagenous stroma
	1	Fibrotic tissue
B2	3	Fibroadenoma
	3	Benign phyllodes
	1	Chronic granulomatous lesion
	1	Inflammatory lesion
B3	4	AIDEP
B4	4	Suspicious for IDC
B5	45	IDC
	1	DCIS
	1	Malignant phyllodes

Histopathological correlation of the subsequent lumpectomy or mastectomy was available for 30 (45%) of the 66 cases. The biopsy diagnoses of these cases were compared with the corresponding histopathology diagnoses, and the ROM for each category was calculated. Table 3 summarizes these findings.

**Table 3:** Comparison of the biopsy diagnosis to their corresponding histopathology diagnosis.

Histopathology diagnosis	B1	B2	B3	B4	B5
Benign	2	5	3	1	0
Malignant	1	0	1	3	13
Total cases with histopathology	3	5	4	4	13
Risk of malignancy	33%	-	25%	75%	100%

The SN, SP, PPV, NPV, and DA were calculated for the groups as follows. In Group 1, the B3, B4, and B5 categories were considered positive. In Group 2, the B4 and B5 categories were considered positive. In Group 3, only the B5 category was considered positive. The maximum SN and NPV were observed when B3, B4, and B5 were considered malignant, but the SP and PPV were low, and the maximum SP and PPV obtained were observed when only B5 was considered malignant. The SN, SP, PPV, NPV, and DA were all relatively high when B4 and B5 were considered malignant (88.8, 90.9, 94.1, 83.3, and 89.6, respectively). Table 4 summarizes these findings.

**Table 4:** Table showing the SN, SP, PPV, NPV, and DA when the cases are divided into three groups. SN, sensitivity; SP, specificity; PPV, positive predictive value; NPV, negative predictive value; DA, diagnostic accuracy.

	Group A (B3, B4, and B5 considered malignant)	Group B (B4 and B5 considered malignant)	Group C (B5 considered malignant)
SN	94.4	88.8	72.2
SP	63.6	90.9	100
PPV	80.9	94.1	100
NPV	87	83.3	68.7
DA	82.7	89.6	82.7

## Discussion

The International Academy of Cytology Yokohama System is widely used for the categorization of fine needle aspiration cytology (FNAC) of breast lesions [2]. The categorization of the diagnosis has been routinely used in the reporting of lesions from breast, thyroid, salivary gland, lymph node, and liver FNAC. However, no such system is commonly used for the reporting of breast core biopsies. In the present study, we assessed the utility of the B categories proposed by the NHSBSP for reporting breast biopsies.

We compared the distribution of cases in each of the B categories with the distribution in studies by Verma et al. [5] and Andreu et al. [3]. Similar to the distribution of cases in our study, which was 65% in the malignant group (B5) and 12% in the benign category (B2), most of the cases (70.39%) in the study by Verma et al. were classified as B5 lesions, while 10% were classified as B2 benign lesions. Only 1.97% of the patients in their study were classified as B3, compared with 4% of the cases in our study. The distribution in the study by Andreu et al. was as follows: B5 with 37.1%; B4 with 0.5%; B3 with 7.6%; B2 with 50.9%, and B1 with 3.9%.

The B1 category indicates when biopsies show predominantly normal elements such as ducts, lobules, stroma, and adipose tissue. Diseases such as focal fibrocystic change (e.g., small islands of apocrine change or microcysts) are usually categorized as B1. Normal lactational change should also be classified as B1 [6]. The B2 category includes conditions such as fibroadenoma, fibrocystic disease, and mastitis. When the diagnosis correlates with the clinical and radiological findings, surgery may not be required [6].

The B3 category is unique, usually including the diagnoses of AIDEPs as well as atypical lobular hyperplasia, radial scarring, papillary lesions, fibroepithelial lesions with hypercellular stroma, mucocele-like lesions, columnar alterations with atypia, and atypical adenosis [3]. In our study, all four cases categorized as B3 involved AIDEPs. A diagnosis of AIDEP is given for atypical intraductal proliferative lesions that do not fulfill the criteria for DCIS or lobular neoplasia. Atypical ductal hyperplasia cannot be diagnosed with certainty by core biopsy because the sample provided may be limited, and the extent of the lesion cannot be determined accurately.

The B4 category is most commonly used in reporting the observation of groups of atypical cells presenting away from the core and small suspicious foci with atypia. A diagnosis of B4 also requires a multidisciplinary meeting to decide on further management strategies (i.e., either repeating the biopsy more deeply or open surgery). The B5b subcategory is mainly used for primary invasive carcinomas of the breast, but should also be used for malignant phyllodes tumors, sarcomas, lymphomas, and metastases to the breast from extra-mammary malignancy.

The histopathological diagnosis of the subsequent mastectomy/lumpectomy was compared with the biopsy diagnosis. Most of the cases reported as benign in histopathology were categorized as B2 based on the core biopsy, and a few cases were categorized as B1 or B3. Most of the cases reported as malignant in histopathology were categorized as B5 on the corresponding core biopsy, though a few cases were categorized as B4. The SN, SP, PPV, NPV, and DA (with B4 and B5 considered malignant) in both our study and the study by Verma et al. [5] were similar. These results are compared in Table 5.

**Table 5:** Comparison of the SN, SP, PPV, NPV, and DA between this study and the study by Verma et al. SN, sensitivity; SP, specificity; PPV, positive predictive value; NPV, negative predictive value; DA, diagnostic accuracy.

	Present study	Verma et al. [5]
SN	88.8%	94.6%
SP	90.9%	100%
PPV	94.1%	100%
NPV	83.3%	33.3%
DA	89.6	94.23%

As shown in Table 6, we compared our findings with those of Andreu et al. [3] and with the NHSBSP’s minimal required standards, including the absolute SN, SP, PPV, and NPV of the B3, B4, and B5 diagnoses [4]. In our study, the SN and SP were 88.8% and 90.9%, respectively. These values are slightly lower than those reported by Andreu et al. and higher than the NHSBSP’s minimum standards.

**Table 6:** Comparison of the present study with the study by Andreu et al. and the minimum NHSBSP standards for quality for the reporting of breast biopsies. NHSBSP, United Kingdom National Health Service Breast Screening Program; SN, sensitivity; SP, specificity; PPV, positive predictive value; FPR, false positive rate.

	Present study	Andreu et al. [3]	NHSBSP minimum standards [4]
Absolute SN	88.8%	90.8%	>92%
SP	90.9%.	94.8%	>75%
PPV (B5)	100%	100%	>99%
PPV (B4)	75%	100%	-
PPV (B3)	25%	16.3%	-
FPR	0.09%	0	<0.2
Rate of missed cancers by core biopsy	5%	5.2%	<5
Suspicious rate (B4/B3)	22%	8.1%	<10%

In our study, the PPV for B5 was 100%, the PPV for B4 was 75%, and the PPV for B3 was 25%. The NPV for B2 was 100%, and the NPV for B1 was 66.6%. The PPV for both B5 and B4 was 100% in the study by Andreu et al., and the PPV in our study exceeded the NHSBSP’s minimum standards [4]. Pinder et al. [7], Rakha et al. [8], and Verschuur-Maes et al. [9] reported a PPV of 20.8% for the B3 category.

The false positive rate (FPR) was 0.09 in our study and 0 in the study by Andreu et al. [3]. The FPRs in our study were lower than the NHSBSP’s minimum standards [4]. The overall rate of missed cancers in our study was 5%, a figure similar to that reported by Andreu et al. and lower than the NHSBSP’s minimum standards [10].

Limitations of the study include a single-center retrospective design and a relatively small sample size. Histopathological correlation was available for only 45% of cases, with a limited representation of B3 spectrum lesions.

## Conclusions

We recommend that breast core biopsy histopathological reports be accompanied by the B code (B1-B5) diagnostic categories proposed by the NHSBSP. This categorization clarifies pathologists’ opinions better than purely descriptive reports and establishes uniformity among pathologists, especially in uncertain cases. The use of these categories thus facilitates the communication between pathologists and clinicians and helps guide patient management. The results reported here also facilitated the analysis of our reports in a manner consistent with the NHSBSP’s quality assurance values. Our core needle biopsy reports are, accordingly, comparable to the minimum recommended standards proposed by the NHSBSP; however, more such studies are necessary to support the need for utilization and comparison with the minimum standards.

## References

[1] Ghoncheh M, Momenimovahed Z, Salehiniya H (2016). Epidemiology, incidence and mortality of breast cancer in Asia. Asian Pac J Cancer Prev.

[2] Ahuja S, Malviya A (2021). Categorization of breast fine needle aspirates using the International Academy of Cytology Yokohama System along with assessment of risk of malignancy and diagnostic accuracy in a tertiary care centre. J Cytol.

[3] Andreu FJ, Sáez A, Sentís M (2007). Breast core biopsy reporting categories—An internal validation in a series of 3054 consecutive lesions. Breast.

[4] Lee AH, Carder P, Deb R, Ellis IO, Howe M, Jenkins JA, Pinder SE (2016). Guidelines for Non-operative Diagnostic Procedures and Reporting in Breast Cancer Screening. https://www.rcpath.org/static/4b16f19c-f7bd-456c-b212f557f8040f66/G150-Non-op-reporting-breast-cancer-screening.pdf.

[5] Verma P, Sharma R, Sharma N, Gulati A, Parashar A, Kaundal A (2021). Fine-needle aspiration cytology versus core-needle biopsy for breast lesions: a dilemma of superiority between the two. Acta Cytol.

[6] Lee AH, Pinder SE (2023). An overview of B coding of breast core biopsy categorisation and management implications. Diagn Histopathol.

[7] Pinder SE, Shaaban A, Deb R (2018). NHS breast screening multidisciplinary working group guidelines for the diagnosis and management of breast lesions of uncertain malignant potential on core biopsy (B3 lesions). Clin Radiol.

[8] Rakha EA, Lee AH, Jenkins JA, Murphy AE, Hamilton LJ, Ellis IO (2011). Characterization and outcome of breast needle core biopsy diagnoses of lesions of uncertain malignant potential (B3) in abnormalities detected by mammographic screening. Int J Cancer.

[9] Verschuur-Maes AH, van Deurzen CH, Monninkhof EM, van Diest PJ (2012). Columnar cell lesions on breast needle biopsies: is surgical excision necessary? A systematic review. Ann Surg.

[10] Ellis IO, Humphreys S, Michell M, Pinder SE, Wells CA, Zakhour HD (2004). Best Practice No 179. Guidelines for breast needle core biopsy handling and reporting in breast screening assessment. J Clin Pathol.

